# Cycling before and after Exhaustion Differently Affects Cardiac Autonomic Control during Heart Rate Matched Exercise

**DOI:** 10.3389/fphys.2017.00844

**Published:** 2017-11-01

**Authors:** Matthias Weippert, Martin Behrens, Anett Mau-Moeller, Sven Bruhn, Kristin Behrens

**Affiliations:** ^1^Institute of Sport Science, University of Rostock, Rostock, Germany; ^2^Department of Orthopaedics, Rostock University Medical Center, Rostock, Germany

**Keywords:** heart rate recovery, fatigue, sudden cardiac death, afferent feedback, central command, ultra short-term heart rate variability, validity, agreement

## Abstract

During cycling before (PRE) and after exhaustion (POST) different modes of autonomic cardiac control might occur due to different interoceptive input and altered influences from higher brain centers. We hypothesized that heart rate variability (HRV) is significantly affected by an interaction of the experimental period (PRE vs. POST) and exercise intensity (HIGH vs. LOW; HIGH = HR > HR at the lactate threshold (HR_LT_), LOW = HR ≤ HR_LT_) despite identical average HR.

**Methods:** Fifty healthy volunteers completed an incremental cycling test until exhaustion. Workload started with 30 W at a constant pedaling rate (60 revolutions · min^−1^) and was gradually increased by 30 W · 5 min^−1^. Five adjacent 60 s inter-beat (R-R) interval segments from the immediate recovery period (POST 1–5 at 30 W and 60 rpm) were each matched with their HR-corresponding 60 s-segments during the cycle test (PRE 1–5). An analysis of covariance was carried out with one repeated-measures factor (PRE vs. POST exhaustion), one between-subject factor (HIGH vs. LOW intensity) and respiration rate as covariate to test for significant effects (*p* < 0.050) on the natural log-transformed root mean square of successive differences between adjacent R-R intervals (lnRMSSD_60s_).

**Results:** LnRMSSD_60s_ was significantly affected by the interaction of experimental period × intensity [*F*_(1, 242)_ = 30.233, *p* < 0.001, η_*p*_^2^ = 0.111]. LnRMSSD_60s_ was higher during PRE compared to POST at LOW intensity (1.6 ± 0.6 vs. 1.4 ± 0.6 ms; *p* < 0.001). In contrast, at HIGH intensity lnRMSSD_60s_ was lower during PRE compared to POST (1.0 ± 0.4 vs. 1.2 ± 0.4 ms; *p* < 0.001).

**Conclusion:** Identical net HR during cycling can result from distinct autonomic modulation patterns. Results suggest a pronounced sympathetic-parasympathetic coactivation immediately after the cessation of peak workload compared to HR-matched cycling before exhaustion at HIGH intensity. On the opposite, at LOW intensity cycling, a stronger coactivational cardiac autonomic modulation pattern occurs during PRE-exhaustion if compared to POST-exhaustion cycling. The different autonomic modes during these phases might be the result of different afferent and/or central inputs to the cardiovascular control centers in the brainstem.

## Introduction

Despite the interest in heart rate (HR) during exercise and recovery, the precise autonomic contributions to HR control across different intensities and exercise modes are still not fully understood, since direct measurements of autonomic outflow to the intact human heart is not feasible (Fisher, 2014). HR itself mainly reflects the chronotropic net effect of the autonomic nervous system. Thus, complementary information and tools are required to get a deeper understanding of the underlying mechanisms of HR control during exercise. Heart rate variability (HRV) is a non-invasive tool that has the potential to quantify autonomic influences on HR (Billman, 2011). However, it should be noted that HR per se profoundly influences HRV (Billman, 2013). Thus, in the current and previous studies (Weippert et al., 2013, 2015b), a HR-matched approach was applied to elucidate autonomic contributions to exercise HR.

Traditionally, the interplay between the sympathetic and parasympathetic nervous system during progressive exercise was thought to be organized in an antagonistic reciprocal fashion, with vagal withdrawal at the beginning of exercise leading to an HR increase of at most 30 beats · min^−1^ followed by an increase in sympathetic activity leading to a further HR acceleration (Robinson et al., 1966). Current data support the view of a continuum of balanced sympathetic-parasympathetic control throughout progressive exercise without clear on/off thresholds (Kannankeril et al., 2004; White and Raven, 2014). Thereby, the influence of both autonomic branches is supposed to be non-linear with a stronger increase of sympathetic and a stronger decrease of parasympathetic contributions at exercise intensities above 60% maximum oxygen consumption and HR > 150 beats · min^−1^ (White and Raven, 2014). Reciprocal antagonism appears to be the dominant mechanism of cardiac autonomic control during orthostatic regulation and many kinds of exercise. However, there are other modes of autonomic control that can generate instantaneous HR under pathological and physiological conditions. Different modulators (e.g., peripheral chemoreceptor, mechanoreceptor, and nociceptor input) can lead to a simultaneous activation (coactivation) of both autonomic branches (Berntson et al., 1991, 1993; Paton et al., 2005, 2006; Fadel and Raven, 2012; Fisher, 2014). Research evaluating the effect of sympathectomy/sympathicolysis in patients with hyperhidrosis speak for a strong cardiac sympathetic-parasympathetic coactivation even under resting conditions (Noppen et al., 1996; Fiorelli et al., 2017). The findings have been linked to a hyperactivity of the sympathetic nervous system during rest in hyperhidrosis, which—also in terms of HR and HRV—can be reversed by this clinical intervention. During physiological conditions, a sympatho-vagal coactivation may allow (i) a greater cardiac output under certain circumstances, such as during the peripheral chemoreceptor reflex and diving responses, as well as (ii) a finer tuning of cardiac function (Paton et al., 2006). Matsukawa (2012) reported that contrary to the traditional idea that vagal withdrawal causes an increase in HR at the onset of exercise, central command did not decrease cardiac vagal efferent activity but allowed sympathetic efferent nerve activity to produce cardiac acceleration in an animal model. Accordingly, recent studies in humans imply that various patterns of autonomic HR control, quantified by HRV, can occur during different exercise modalities (Gonzalez-Camarena et al., 2000; Weippert et al., 2013, 2015b). For example, a significantly different HRV pattern has been shown in response to dynamic and static exercise (Weippert et al., 2014, 2015b) or during dynamic upper and lower body exercise (Leicht et al., 2008) despite similar net effects on HR itself. Different feedback from baro-, metabo-, and mechanosensitive fibers as well as changes in central command and “effort sense” might contribute to these distinct autonomic cardiovascular adjustments during exercise (Boushel, 2010; Matsukawa, 2012; Fisher, 2014; Ichinose et al., 2014).

The main goal of this study was to assess whether autonomic HR control, mirrored by HRV, differs between cycling before volitional exhaustion (PRE) and cycling at a low workload immediately after exhaustion (POST). It was assumed that sympathetic-parasympathetic coactivation is more pronounced during early POST compared to HR-corresponding PRE periods, probably due to a strong vagal rebound following reduced central command and maintained sympathetic activity stimulated by metabo- and baroreceptors. Therefore, five adjacent 60 s-segments of the active recovery period immediately after peak exercise (POST 1–5) were matched with their HR-corresponding 60 s-segments during progressive exercise before exhaustion (PRE 5–1). It was hypothesized that the pattern of autonomic HR modulation, is significantly affected by (i) the experimental period (PRE vs. POST exhaustion) and (ii) the exercise intensity (HIGH vs. LOW intensity) despite identical net effects on average HR. Exercise intensity was dichotomized into LOW [ = HR ≤ HR at the individual lactate threshold (HR_LT_)] and HIGH (= HR > HR_LT_). HR-matched PRE and POST exhaustion periods at HIGH or LOW intensity served as a physiological model for states of different afferent and central inputs to the cardiovascular control center in the Medulla oblongata.

Autonomic HR control was evaluated by the analysis of HR as well as of the root mean square of the successive differences (RMSSD_60s_) of adjacent inter-beat intervals (R-R) over a period of 60 s. It is supposed, that RMSSD_60s_ mirrors predominantly parasympathetic HR modulation (Task Force of the European Society of Cardiology the North American Society of Pacing and Electrophysiology, 1996; Goldberger et al., 2006).

Before we tested the above-mentioned hypothesis, we assessed the internal and external validity of ultra-short-term HRV (= 60 s-segments). Traditionally, short-term evaluation of HRV bases on the analysis of 180–300 s-segments; however, these time windows are not appropriate to give a representative picture of transient processes like recovery from exercise. Validity and reproducibility of HRV ultra-short measures have been tested under resting conditions and recovery from exercise (Thong et al., 2003; Nussinovitch et al., 2011; Esco and Flatt, 2014; Munoz et al., 2015; Nakamura et al., 2017); however, a paucity of research exists regarding the validity and agreement with standard short-term measures during low to high intensity exercise. Therefore, agreement of natural log-transformed 60 s-RMSSD (lnRMSSD_60s_), -high frequency power (lnHFP_60s_), and -low frequency power (lnLFP_60s_) of the R-R spectra with their respective traditional short-term HRV indices, calculated for a time interval of 180 s (lnRMSSD_180s_, lnHFP_180s_ and lnLFP_180s_), were assessed across different exercise intensities. In addition, lnRMSSD_60s_ was plotted against the corresponding HR during incremental exercise. The aim was to correlate the behavior of lnRMSSD_60s_ during incremental cycling with the current model of parasympathetic-sympathetic HR control during progressive exercise (White and Raven, 2014).

In conclusion, this paper comprised two studies: (i) a validation study and (ii) a comparison of HRV during HR-matched cycling before and after volitional exhaustion. The validation study (i) was a prerequisite to the second experiment and aimed to fill the gap of knowledge regarding the agreement of ultra-short-term measures of HRV with their respective measures of traditional recording length during various exercise intensities. The comparison study (ii) aimed to elucidate the mechanism of autonomic HR-control during conditions of distinct afferent and central inputs to the cardiovascular control center in the medulla oblongata. Therefore, HRV was compared between exercise and active recovery segments of corresponding net HR.

## Material and methods

### Ethics statement

This study was performed in compliance with the Declaration of Helsinki and approval of the local ethics committee at Rostock University was obtained. All participants gave their written informed consent to take part.

### Validation study

Twenty participants (10 females, 22.8 ± 2.2 years, 62.0 ± 5.3 kg, 169.2 ± 5.8 cm; 10 males, 23.9 ± 1.3 years, 76.1 ± 6.6 kg, 182.5 ± 5.9 cm) volunteered in this study. All participants were non-habitual smokers, free of medication and abstained from any exhaustive exercise and alcohol for >24 h prior to the experiment. Furthermore, the consumption of caffeine or nicotine was not allowed during the night and on the morning of the experiment. After a medical clearance participants performed an incremental cycling test (ER 900, Ergoline, Germany) until volitional exhaustion (initial load 30 W + 30 W 5 · min^−1^) at a constant pedaling rate (60 revolutions · min^−1^). Beat-to-beat HR (S810i, Polar, Finland) was continuously measured throughout the test. Individual R-R recordings were screened for five stationary 180 s-segments that covered a wide physiological activity range from low to maximal HR. HRV analysis of the resulting 100 R-R interval segments was performed using the free software Kubios HRV 2.2 (University of Kuopio, Finland). All analyzed R-R recordings exhibited low noise (rate of erroneous R-R intervals below 5%). Before the computation, R-R time series were detrended and corrected for artifacts using adaptive filtering. Frequency analysis was performed using a Fast Fourier Transform (Welch's periodogram: 256 s-window with 50% overlap). Internal validity was assessed for the parasympathetic HRV indices lnHFP and lnRMSSD as well as for lnLFP—mirroring both parasympathetic and sympathetic effects on HR (Task Force of the European Society of Cardiology the North American Society of Pacing and Electrophysiology, 1996; Goldberger et al., 2006; Smith et al., 2013). Agreement of the 180 s- with the 60 s-HRV indices was assessed using estimation equation, mean and standardized mean bias, typical error of estimate, and validity correlation. Statistical indices were calculated and interpreted according to Hopkins (2015). In a second step, lnRMSSD_60s_ was plotted against the corresponding HR to visualize the behavior of the vagally mediated HRV during incremental exercise.

### Comparison of HRV during HR-matched cycling before and after exhaustion

Fifty healthy participants (13 females, 25.7 ± 5.8 years, 65.1 ± 10.6 kg, 169.7 ± 5.3 cm; 37 males, 26.4 ± 7.1 years, 77.9 ± 9.6 kg, 182.6 ± 6.3 cm) volunteered in this study. Inclusion criteria as well as load protocol are identical with and described under *(i) validation study*. Immediately after exhaustion, workload was reduced to 30 W and participants kept pedaling at 60 revolutions · min^−1^ for additional 10 min (active recovery = POST). Beat-to-beat HR (S810i, Polar, Finland), oxygen uptake (VO_2_), and carbon dioxide output (VCO_2_) (EOS Sprint, Jaeger, Germany) were continuously recorded during the test. Blood lactate concentration was determined from capillary blood samples (LactateScout, SensLab, Germany), drawn from the left ear lobe at the end of each stage. Cardiorespiratory data and blood lactate concentration at exhaustion are described in Table 1. HR_LT_ was determined at the lowest value of the lactate-equivalent (ratio of blood lactate concentration and VO_2_) during the incremental test (Dickhuth et al., 1999). For the statistical testing, exercise intensity was dichotomized into LOW [ = HR ≤ HR at the individual lactate threshold (HR_LT_)] and HIGH (= HR > HR_LT_) for both, PRE and POST, periods.

**Table 1:** Cardiorespiratory data and blood lactate concentration at exhaustion (*N* = 49).

	**Mean (*SD*)**
Heart rate (beats · min^−1^)	185.2 (13.4)
Relative VO_2_ (ml · min^−1^ · kg^−1^)	43.0 (8.9)
Ventilation (L · min^−1^)	103.5 (25.3)
Respiratory exchange ratio	1.2 (0.1)
Lactate concentration (mmol · L^−1^)	9.7 (2.7)

**Figure 1:**
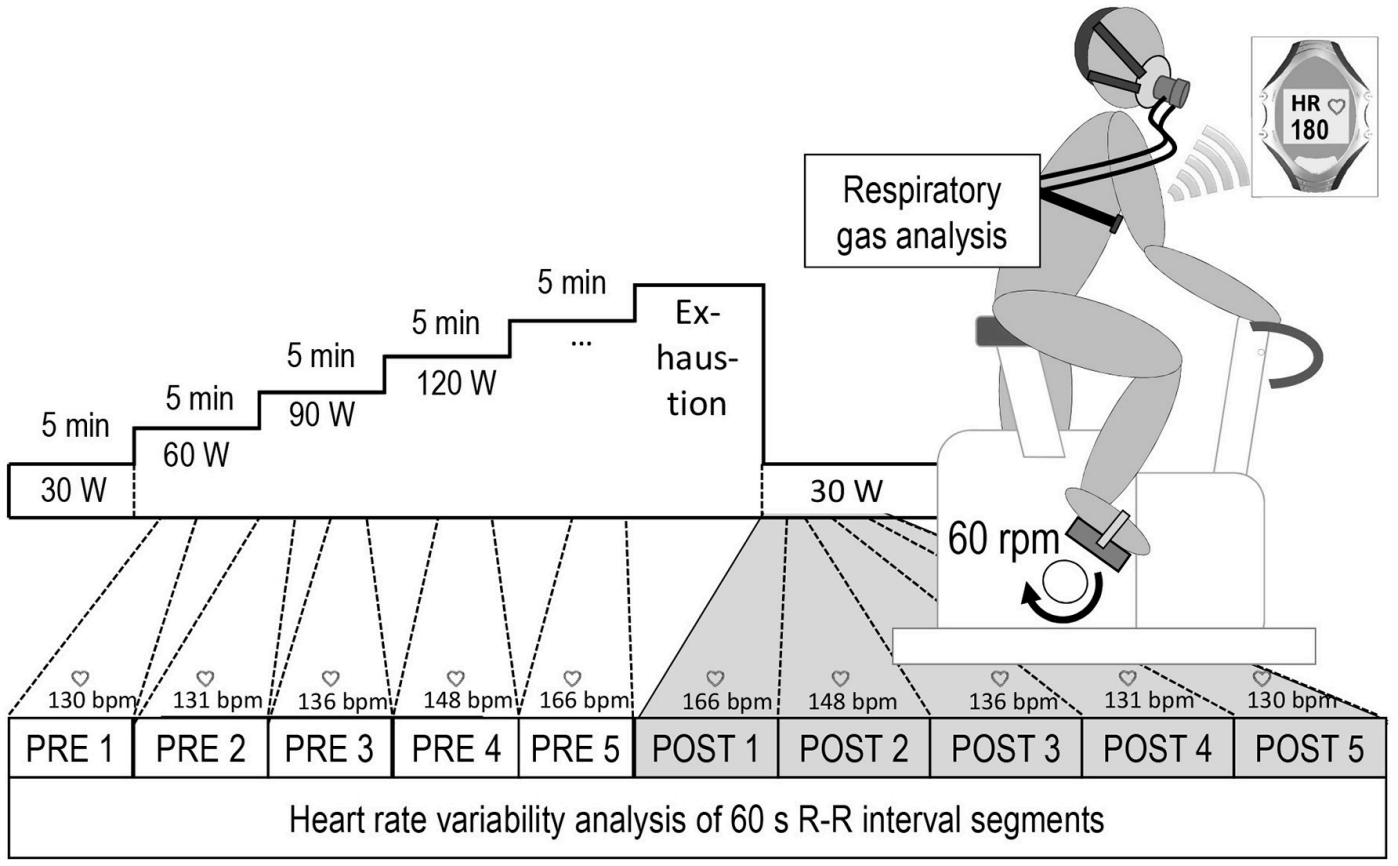
Overview of the experimental setup and the heart rate based data matching procedure, POST 1–5 = five consecutive 60 s-segments during active recovery from cycling until exhaustion, PRE 5–1 = corresponding HR-matched 60 s-periods during exercise.

Due to measurement artifacts, data of one male subject had to be excluded from further analysis. For all other participants (*N* = 49), the first five adjacent 60 s-segments during the immediate active recovery (POST 1–5) were matched with corresponding 60 s-segments yielding the same average HR during the incremental cycling test. Thus, POST 1 was matched with PRE 5, POST 2 with PRE 4 and so on (Figure 1). All analyzed R-R interval recordings exhibited low noise (rate of erroneous R-R intervals below 5%). LnRMSSD_60s_ was used to quantify vagal HR modulation. Before the computation, R-R time series were detrended and corrected for artifacts using adaptive filtering (Kubios HRV 2.2, University of Kuopio, Finland). Bland-Altman Plots (Bland and Altman, 2007) and Student's *t*-test statistics were applied to statistically verify the agreement of the matched R-R segments. A repeated-measures analysis of variance (IBM SPSS Statistics 22.0, USA) was carried out to test the effect of measurement time, experimental period (PRE vs. POST) and their interaction on lnRMSSD. To test the hypothesis of exercise intensity being a significant contributor to distinct HRV patterns at similar HR levels, experimental period was used as repeated measures factor (PRE vs. POST) and intensity as between subject factor (HIGH vs. LOW). Additionally, high frequency peak values (HF_peak_), a measure of respiration rate (Thayer et al., 2002), was entered as covariate to control for potential bias. If data violated the assumption of sphericity, Greenhouse-Geisser corrected *p*-values and respective degrees of freedom were reported. For *post-hoc* pair-wise comparisons significance levels were adjusted using Bonferroni's correction.

## Results

### Validation study

**Figure 2:**
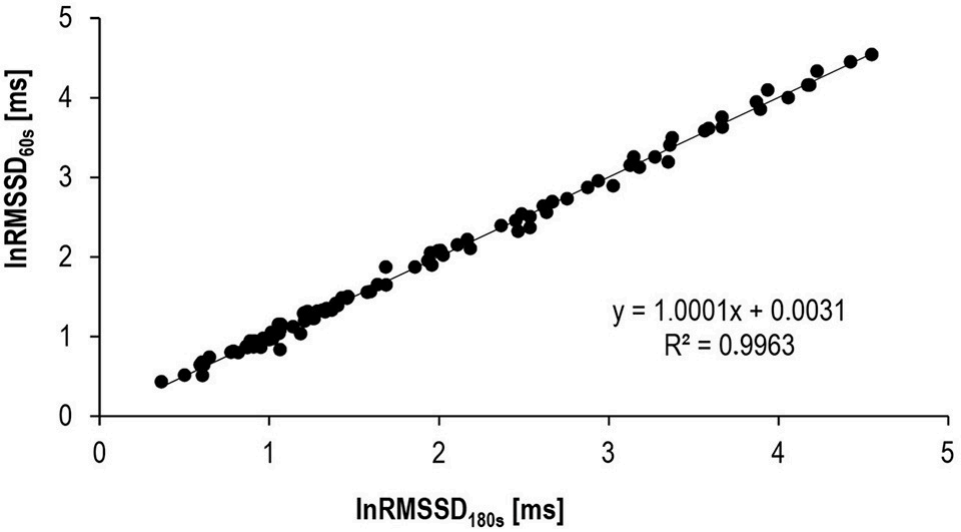
Agreement between lnRMSSD_60s_ and traditional lnRMSSD_180s_.

Results of the internal validation study are provided in Table 2 and Figure 2. Mean biases between HRV_180s_ and HRV_60s_ as well as typical errors were very low and the correlations between the very short and the traditional short-term HRV very high.

**Table 2:** Validation indices and interpretation of standardized mean bias and standardized typical error for ultra-short-term HRV indices (60 s) in comparison to traditional short-term HRV (180 s).

**HRV_60s_ compared to HRV_180s_**	**Mean bias (standardized)**	**Typical error of estimate (standardized)**	**Validity correlation Pearson's *r* (lower CL/upper CL)**	Interpretation of standardized mean bias/typical error^*^
lnRMSSD	0.00 ms (0.00)	0.07 ms (0.06)	1.00 (1.00/1.00)	Trivial/Trivial
lnLFP	−0.08 ms^2^ (−0.03)	0.49 ms^2^ (0.16)	0.99 (0.98/0.99)	Trivial/Small
lnHFP	−0.03 ms^2^ (−0.01)	0.42 ms^2^ (0.13)	0.99 (0.99/0.99)	Trivial/Small

* *< 0.1 trivial, 0.1-0.3 small, 0.3-0.6 moderate*.

*CL, confidence limits; lnRMSSD, natural log-transformed root mean square of successive differences between adjacent R-R intervals; lnHFP, natural log-transformed high frequency power of the HRV spectrum; lnLFP, natural log-transformed low frequency power of the HRV spectrum*.

LnHFP_60s_, lnLFP_60s_, and lnRMSSD_60s_ showed high agreement with their respective traditional short-term 180 s-HRV indices. Confidence limits of the validity correlation coefficients *r* were between 0.98 and 1.00.

**Figure 3:**
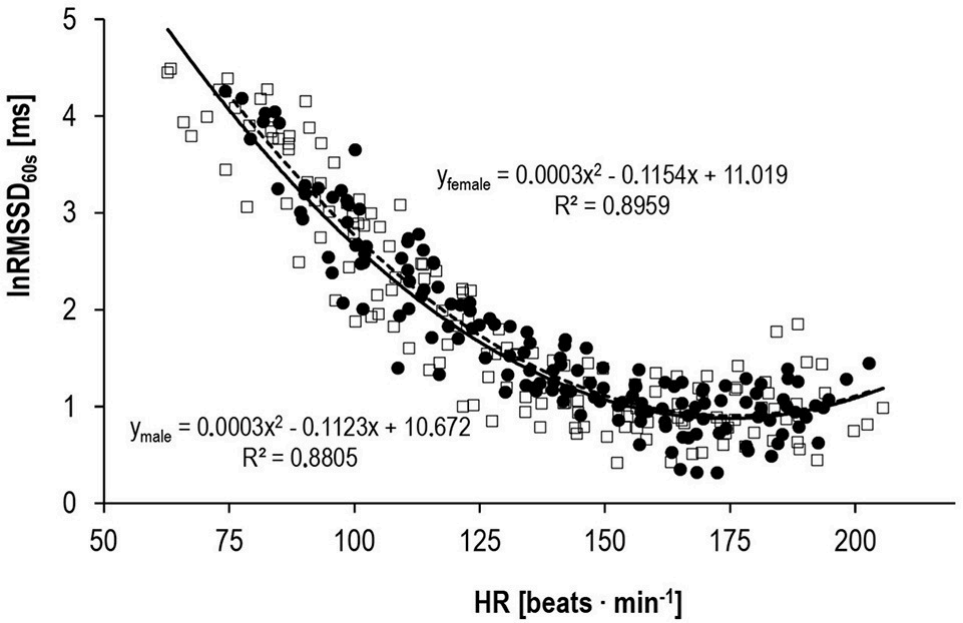
Correlation between lnRMSSD_60s_ and HR for male (□, solid line, *n* = 10) and female subjects (•, dashed line, *n* = 10) during progressive exercise.

Results of the external validity approach showed a three-phase behavior for lnRMSSD_60s_. It almost linearly decreased to a minimum, plateauing at HR-values around 170 beats · min^−1^, followed by a small rebound close to peak exercise (Figure 3).

### Comparison of HRV between HR-matched cycling before and after exhaustion

**Figure 4:**
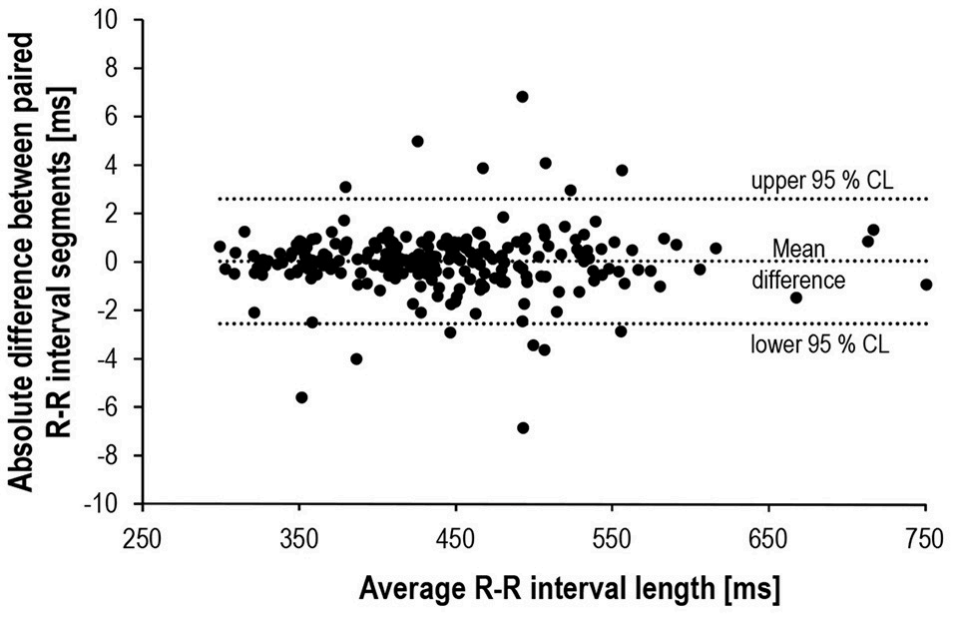
Bland-Altman plot of the absolute R-R differences (*N* = 245) between matched pairs (PRE vs. POST).

**Figure 5:**
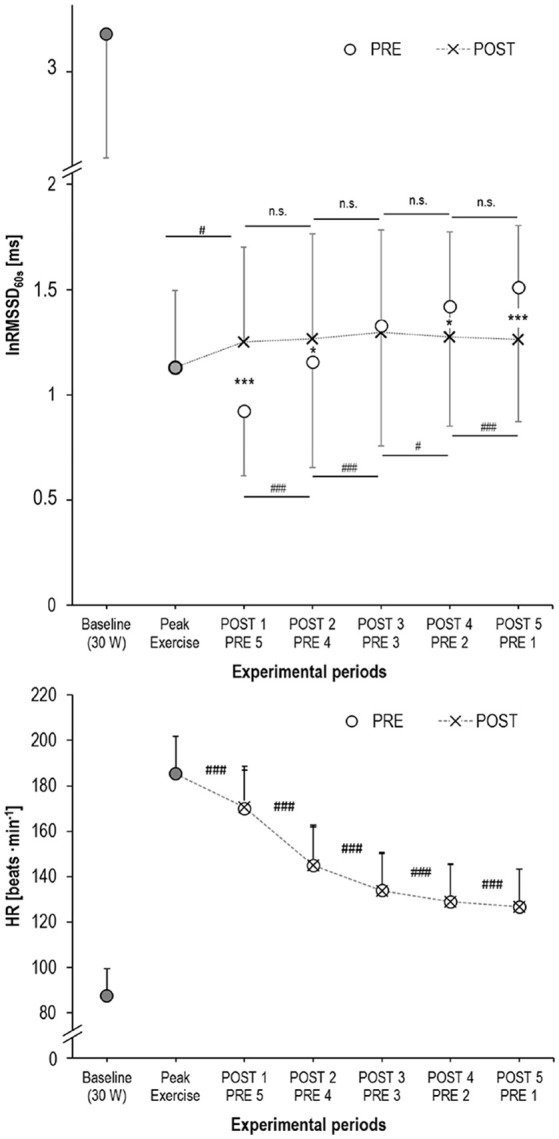
Mean and standard deviation of lnRMSSD_60s_ (upper panel) and HR (lower panel) at baseline (cycling with 30 W, filled circles), at peak exercise (filled circles), during active recovery from exhaustion (POST, crosses) and the HR-matched segments during cycling before exhaustion (PRE, open circles); significance of the lnRMSSD_60s_-differences: ^***^p < 0.001, ^*^p < 0.050 between PRE vs. POST; ###*p* < 0.001, #*p* < 0.050 between adjacent measurement time points.

Bland-Altman plot and *t*-test statistics confirmed the high correlation (*r* = 1.0, *p* < 0.001) and agreement (mean difference: 0.02 ± 1.29 ms, *p* = 0.792) between matched R-R segments, thus verifying the validity of the experimental approach (Figure 4). Despite identical HR for the matched R-R segments, lnRMSSD_60s_ was significantly affected by an interaction of measurement time × experimental period [*F*_(4, 192)_ = 30.733, *p* < 0.001, η_*p*_^2^ = 0.390]. Figure 5 illustrates the behavior of HR and lnRMSSD_60s_ during cycling at baseline, peak exercise, and during PRE and POST exhaustion cycling. HR and lnRMSSD_60s_ at peak exercise were significantly higher (*p* < 0.001) and lower (*p* = 0.017), respectively, if compared to the first minute of POST. While HR further decreased from POST 1 to POST 5, no additional rebound from POST 1 to POST 5 was evident for lnRMSSD_60s_. In contrast, lnRMSSD_60s_ progressively decreased with increasing HR during PRE. Both HR and lnRMSSD_60s_ did not reach baseline values after 5 min of recovery (POST 5).

**Figure 6:**
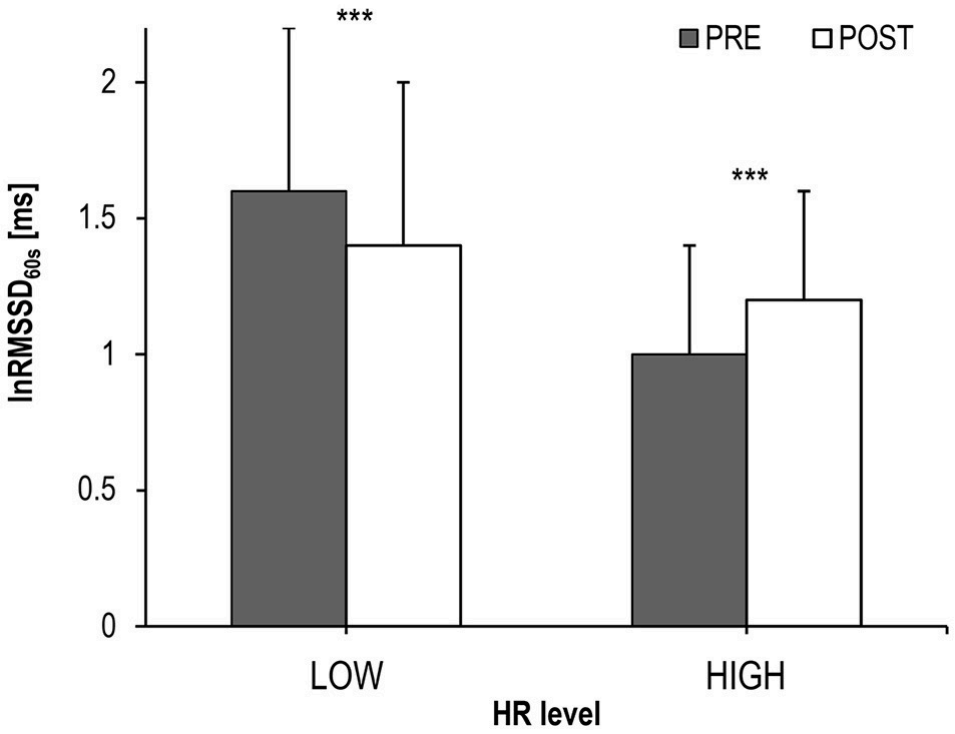
LnRMSSD_60s_ (Mean and *SD*) during cycling before (PRE) and after exhaustion (POST) at HIGH (HR > individual lactate threshold HR) and LOW (HR ≤ individual lactate threshold HR) intensity; significance of the lnRMSSD_60s_-differences between PRE vs. POST: ^***^p < 0.001.

Individual workload responses during the different experimental periods may not be perfectly comparable between subjects due to differences in aerobic capacity, training state and individual recovery behavior. Therefore, metabolic state—estimated by individual lactate threshold—was used as a factor potentially influencing the behavior of lnRMSSD_60s_. ANCOVA revealed that—despite no interaction effect on average R-R interval [*F*_(1, 242)_ = 1.888; *p* = 0.665; ņ_*p*_^2^ = 0.001]—lnRMSSD_60s_ was affected by the interaction of experimental period × intensity [*F*_(1, 242)_ = 30.233, *p* < 0.001, ņ_*p*_^2^ = 0.111; Figure 6].

## Discussion

Aim of this study was twofold: (i) validity of ultra-short-term HRV measures against traditional HRV was tested across a broad range of exercise intensities and (ii) autonomic HR modulation, assessed by HRV, was compared during different exercise conditions.

### Validation study

Our data confirmed the internal validity of the 60 s HRV indices lnRMSSD_60s_, lnLFP_60s_, and lnHFP_60s_ compared to their respective 180 s indices. LnRMSSD_60s_ performed even better than the frequency domain indices. Based on the standardized mean bias as well as the standardized mean error, the difference between lnRMSSD_60s_ and lnRMSSD_180s_ was rated trivial. Thus, very short-term lnRMSSD_60s_ can be used interchangeably with traditional lnRMSSD_180s_ data segments. Furthermore, the behavior of lnRMSSD_60s_ during progressive exercise in our study was coherent with the current model of sympathetic-parasympathetic interaction during progressive exercise. According to this model and empirical data, vagal modulations of HR should be detectable until submaximal workloads around 150 · beats min^−1^ (Ng et al., 2009; White and Raven, 2014). Thus, our results indirectly speak for the external validity of lnRMSSD_60s_ and its potential to reflect these vagal effects on HR control during submaximal exercise. Previous autonomic blocking experiments have also provided direct evidence for lnRMSSD_60s_ being a valid tool to describe parasympathetic reactivation following exercise (Goldberger et al., 2006).

### Comparison of HRV between HR-matched cycling before and after exhaustion

The behavior of HR and lnRMMSD_60s_ during the final minute of cycling at maximum workload and during POST 1 indicated a strong parasympathetic reactivation immediately after peak exercise. In our experiment, RMSSD_60s_ plateaued at this increased level throughout the following POST periods. This is in contrast to the findings of Goldberger et al. (2006) who found RMSSD to progressively increase across the first 3 min of recovery after a shorter submaximal exercise protocol, but in line with other studies that have shown a slower recovery of vagally mediated HRV after maximal intensity exercise (see Michael et al. (2017) for review). Assuming that cardiac vagal activity plateaus on a high level as suggested by lnRMSSD_60s_, the progressive HR decrease during POST can be mainly attributed to sympathetic withdrawal.

The main finding of an interaction effect of experimental period (PRE vs. POST exhaustion cycling) × intensity (LOW vs. HIGH) confirmed the hypothesis of a different HRV pattern during these kinds of exercise, despite an identical net effect on average R-R interval length. We cautiously conclude that lnRMSSD reflects a different chronotropic pattern generated by the autonomic network in the medulla oblongata, which arises from differences in central command and afferent feedback during the different experimental periods. When HR is still above HR_LT_ during early POST, vagal HR modulation is stronger compared to cycling at corresponding HRs during PRE. Thus, during the very first minutes after exhaustive exercise, net HR might result from a stronger sympathetic-parasympathetic coactivation if compared to high intensity cycling at similar HR (Berntson et al., 1991, 1993). An alternative explanation for a decreased vagally modulated HRV during late PRE might be a high or even saturated sympathetic tone (White and Raven, 2014). However, because direct nerve recordings in the intact human heart are impracticable and HRV reflects only the end-organ response that integrates different (autonomic) facilitatory and inhibitory effects on HR, the underlying mechanisms remain speculative.

Several neural feedback and feedforward mechanisms and their interaction may play a role in generating the distinct HRV pattern during HR-matched PRE and POST periods. The reactivation of vagal activity during early POST might counteract or even overpower the potential sympathetically mediated HR elevation by the metaboreflex (Fisher, 2014). The vagal rebound may occur due to the removal of inhibitory inputs from central command and the muscle mechanoreceptors and/or baroreflexes (Fisher, 2014 and references cited herein). This model is consistent with our results and the assumption of a pronounced sympathetic-parasympathetic coactivation during early POST.

Principally, afferent signaling from several types of receptors, including peripheral and central chemoreceptors, arterial baroreceptors, metabolite-, mechano-, and nocisensitive afferents within and around the working muscle (Fisher, 2014; Amann et al., 2015; Michelini et al., 2015) should profoundly differ during PRE and POST. For example, baroreceptor inputs change by the rapid decline of arterial blood pressure upon cessation of heavy exercise, inter alia by a decreased venous return due to a reduced activity of the skeletal muscle pump during POST (Takahashi et al., 2005). Along with decreases in central command at the beginning of POST, the cardiac baroreflex sensitivity increases via an augmented vagal activity (Ogoh et al., 2002, 2005; Gallagher et al., 2006). An alteration of the baroreflex sensitivity might in turn affect HRV (Di Rienzo et al., 1991). Additionally, altered feedback from muscle mechano- and metabolite-sensitive afferents during POST compared to PRE might contribute to different ANS outflow to the heart (Gladwell et al., 2005; Amann et al., 2010; Fisher, 2014). Mechanoreceptor inputs to the cardiovascular control centers in the brainstem decrease due to lesser muscular engagement during active recovery and would thereby alter cardiac autonomic drive (Crisafulli et al., 2003; Trinity et al., 2010). A stronger phasic compromised muscle blood flow in the exercising muscles (Holtz, 1996) during PRE might have also contributed to a distinct activity pattern of metabolite-sensitive afferents, resulting in a different autonomic HR modulation during PRE and POST (Alam and Smirk, 1938; O'Leary, 1993; Spranger et al., 2013; Fisher, 2014; Amann et al., 2015). Altered feedback from central and peripheral chemoreceptors during PRE and POST periods might have also modulated cardiac control. During mild to moderate dynamic exercise, arterial CO_2_ pressure slightly increases toward hypercapnic tension, whereas it returns to resting values or even hypocapnic tension during heavy to exhaustive exercise due to the respiratory compensation of acidosis (Dempsey, 1988; Forster and Pan, 1988; Raven et al., 2013). The compensatory hyperventilation during heavy exercise can result in a respiratory alkalosis in the cerebral fluid that is expected to reduce central chemoreceptor discharge and to stimulate neural projections that inhibit the carotid afferent chemosensitive activity (Whipp and Ward, 1998). Taken together, it can be assumed that a different chemical milieu of the arterial blood and/or cerebral fluid during the matched PRE and POST leads to an altered signaling from peripheral and/or central chemosensitive afferents to the cardiovascular control centers in the medulla oblongata. Despite chemoreflexes being powerful modulators of the neural ventilatory and circulatory control, they are itself subject to important negative feedback interactions with baro- and pulmonary stretch receptors as well as to modulatory effects of circulating catecholamines (Kara et al., 2003; Mansukhani et al., 2015). The inhibitory effect of the baro- and pulmonary stretch reflexes in turn depends on arterial CO_2_-pressure (Somers et al., 1989, 1991), which—in turn—depends on exercise intensity and the ventilatory response.

## Limitations

Despite indirectly controlling for different respiratory rates by using the HF_peak_ from the HRV spectrum as a covariate, it cannot be fully neglected that ventilation has differently affected HRV during the two experimental periods. However, previous studies that systematically and profoundly manipulated breathing pattern, have shown that RMSSD is robust against different breathing patterns at physiological respiration rates >0.1 Hz (Schipke et al., 1999; Weippert et al., 2015a). Thus, a significant impact of breathing on RMSSD is not very likely, especially since breathing rates did not profoundly differ between the matched experimental periods. It has generally to be considered that RMSSD is not a direct measure of vagal tone. Finally, alterations in circulating plasma catecholamines during PRE and POST (Hagberg et al., 1979) may have also slightly affected HRV via direct effects on the sinus node (Kienzle et al., 1992; Breuer et al., 1993; Grossman and Taylor, 2007). One strength of this study is also its weakness: because we measured effects on HR control during physiological conditions without artificially manipulating afferent feedback and/or pharmacological blocking of parasympathetic or sympathetic receptors, conclusions regarding the precise contributions of afferent feedback and central command modulation on autonomic HR control remain elusive.

## Summary

Our experiment evidences a different HRV during HR-matched cycling before and after exhaustion that is also dependent on HR level in healthy subjects free of cardiovascular diseases. The HRV results speak for distinct patterns of autonomic neural drive to the heart during these conditions, exerting a similar net effect on HR (Berntson et al., 1991, 1993). The different autonomic control pattern might be the result of complex and distinct interactions of afferent feedback and/or central command during PRE and POST periods (Table 3). Whether this differential HRV response during and after exercise is also evident in subjects with cardiovascular diseases remains to be investigated. However, since ANS function may play a role in exercise related cardiovascular events like sudden cardiac death (Thompson et al., 2007; Franciosi et al., 2017), a different response pattern or amplitude in persons at risk is not unlikely.

**Table 3:** Potential feedback and feedforward contributions to autonomic cardiac control during PRE and POST at high and low HR level, respectively.

**Heart rate level**	**High (**>**HR**_**LT**_**)**		**Low (**<**HR**_**LT**_**)**	
**Exercise condition**	**PRE**	**POST**	**PRE**	**POST**
Central command	↑↑	↓↓	↑	↓↓
Metabosensitive afferents from muscles	↑	↑↑	↓	↑
Mechanosensitive afferents from muscles and joints	↑↑	↓↓	↑	↓↓
Baroreceptor afferents	↑↑	↓↓	↑	↓
Central and peripheral chemoreceptors	↔/↓/↑ (?)	↑	↑/↔ (?)	↔/↑ (?)

*HR_LT_, heart rate at lactate threshold; PRE, cycling before exhaustion; POST, cycling after exhaustion*.

*Please note that (a) arrows indicate relative increases/decreases compared to the respective condition; (b) interactions are complex and potentially non-linear, e.g., feedback from central and peripheral chemoreceptors (e.g., during hypocapnic arterial hypoxia) might have opposing effects on autonomic neural drive; (c) complementary feedback from other receptors (e.g., pulmonary stretch afferents or nociceptors) might modulate the cardiac response to exercise*.

## Author contributions

MW, MB, and KB designed this study; MW, collected, analyzed, and interpreted the data; MW drafted the manuscript, all authors revised the manuscript and approved the final version to be published.

### Conflict of interest statement

The authors declare that the research was conducted in the absence of any commercial or financial relationships that could be construed as a potential conflict of interest.
